# A review of linc00673 as a novel lncRNA for tumor regulation

**DOI:** 10.7150/ijms.48134

**Published:** 2021-01-01

**Authors:** Kunjie Zhu, Zhaojian Gong, Panchun Li, Xianjie Jiang, Zhaoyang Zeng, Wei Xiong, Jianjun Yu

**Affiliations:** 1 Department of Head and Neck Surgery, Hunan Cancer Hospital and The Affiliated Cancer Hospital of Xiangya School of Medicine, Central South University, Changsha, Hunan, China.; 2 NHC Key Laboratory of Carcinogenesis, and Key Laboratory of Carcinogenesis and Cancer Invasion of the Chinese Ministry of Education, Cancer Research Institute, Central South University, Changsha, Hunan, China.; 3 Department of Oral and Maxillofacial Surgery, The Second Xiangya Hospital, Central South University, Changsha, Hunan, China.

**Keywords:** LncRNA, Diagnosis, Prognosis, SNP, Cancer

## Abstract

Long non-coding RNAs (LncRNAs) act as regulators and play important roles in a variety of biological processes. These regulators constitute a huge information network among genes and participate in the pathophysiological process of human diseases. Increasing evidence has demonstrated that LncRNA, as an oncogene or tumor suppressor gene, is closely related to the occurrence and development of tumors. Linc00673 is a recently discovered LncRNA molecule that is dysregulated in several solid tumors. Moreover, its genetic polymorphism is believed to affect the susceptibility of a population to the corresponding cancer species. This article summarizes the role of Linc00673 in different human cancers and its molecular mechanisms with a focus on the characteristics of Linc00673 and the existing literature on it while highlighting the future research directions for Linc00673. Linc00673 has the potential to become a feasible clinical diagnostic and prognostic marker toward providing a new molecular therapeutic target for cancer patients.

## Introduction

Cancer is a malignant disease caused by the transformation of normal cells into cancer cells. The key initiation events for the transformation include oncogene activation, telomerase deletion, and aneuploidy induction 1, 2. The early human genome project studied genes that are abundantly expressed, evolutionarily conserved, and can encode proteins. Hence, RNAs that did not encode proteins were treated as the “dark matter” of genome 3, 4. However, the rapid development of new sequencing technologies such as tiled arrays and high-throughput sequencing has highlighted that >98% of the RNAs transcribed from genes in the human cells are non-coding RNAs 5. Long non-coding RNAs (LncRNAs) are longer than 200 nt and are produced by RNA polymerase ΙΙ. They can regulate the gene expression at epigenetic, transcriptional, and post-transcriptional processing levels by acting on the nucleic acid molecules and proteins by cis or trans 6, and they participate in a series of cell events such as differentiation, proliferation, invasion, apoptosis, and migration 7.

Linc00673 is located on the chromosome 17q24.3 8, at approximately 275-kb telomeric of SOX9 9. This LncRNA sequence contains a conserved region with significant similarity to steroid receptor RNA activator 1 (SRA1) and is hence also known as “SRA-like non-coding RNA” (SLNCR) 10. A 2015 article 11 was the first one to grab the attention of scientists from all across the world to Linc00673. This article reported about single nucleotide polymorphisms (SNPs) associated with pancreatic cancer susceptibility in individuals of European descent; two of these SNPs are located on Linc00673 11. Subsequent research on Linc00673 covered diverse cancer types. In the present paper, we have summarized the correlation between Linc00673 and clinical factors in different cancer types. Simultaneously, we have focused on the molecular mechanism of Linc00673 in regulating tumors, as well as discussed the prospects of clinical application of Linc00673. Finally, we have proposed the possible future research directions for Linc00673.

## Linc00673 expression in tumor tissues as a molecular marker

Over the past few years, pan-oncogenic transcriptome analysis has been performed after thorough rearrangement and relabeling of thousands of RNA sequences retrieved from The Cancer Genome Atlas (TCGA). Reportedly, LncRNAs are often dysregulated in tumors and are associated with other cancer-related genetic modifications 12. These LncRNAs, as oncogenes or tumor suppressors, are often involved in the regulation of malignant tumors. The current clinical research on Linc00673 covers a range of cancer types, including lung cancer 13-15, pancreatic cancer 9, 16, 17, gastric cancer 18, 19, breast cancer 20-22, liver cancer 23, thyroid cancer 24, 25, prostate cancer 26, ovarian cancer 27, tongue cancer 28, and colorectal cancer 29. Our findings suggest that the Linc00673 expression is strongly related to the baseline clinical characteristics of patients, such as tumor size, lymph node metastasis, and the TNM Classification of Malignant Tumors stage (**Table 1**). Moreover, a few studies used normal tissues as control to draw the receiver operating characteristic curve. As a result, the areas under the curve of Linc00673 in lung cancer 13 and pancreatic cancer 17 were found to be 0.683 and 0.6093, respectively. In addition to the tumor tissues, the corresponding trend of Linc00673 expression was also detected in the peripheral blood 17, 30. This study report suggests that Linc00673 may be a clinically effective biomarker for early molecular diagnosis. In addition, the overall survival of patients is an effective indicator to evaluate the clinical significance of the differential expression of LincRNA. Cancer patients can be divided into 2 groups based on the expression of Linc00673: high and low. The Kaplan-Meier survival analysis revealed that the high expression of Linc00673, which acts as a pro-oncogene, is related to adverse outcomes for patients 14, 17-19, 21-24, 27-29. The relationship between the Linc00673 expression and clinical outcomes of cancer patients suggests that the expression level may be used as a potential prognostic indicator. The expression and related clinical characteristics of Linc00673 in different cancer tissues are presented in **Table 1.**

## Mechanism of Linc00673 in Cancers

### rs11655237 of Linc00673 increases the population's susceptibility to cancer

**Figure 1:**
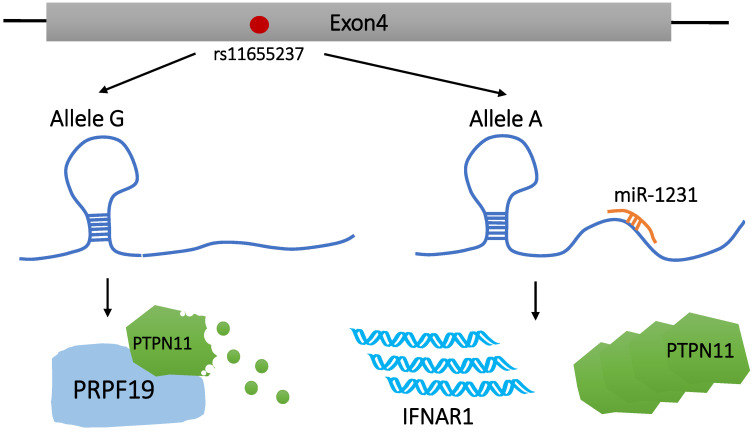
Effect of rs116552337 G > A on Linc00673 function. rs116552337 G > A resulted in the formation of miR-1231-recognition binding region on Linc00673, which ultimately led to the upregulation of IFNAR1 expression and the accumulation of PTPN11.

Genome-wide association study (GWAS) has identified thousands of phenotype-related DNA sequence variants toward investigating potential reasons for phenotypic differences and disease susceptibility among individuals 31, 32. SNPs are one of the most common heritable mutations that induce DNA sequence polymorphisms at the gene level, accounting for >90% of all known polymorphisms 33. There are 4 different variations of SNPs, namely, transitions, transversion, deletion, and insertion. Genetic mutations in protein-coding regions are often harmful or fatal, and the disease risk-related SNPs identified by GWAS are mostly located in the non-coding regions 34. These SNPs can affect the regulation of LncRNAs via modification of the LncRNA sequence or expression level. These modifications are not immediately fatal, but can enhance susceptibility in carriers to a variety of diseases, including tumors 34, 35. Until date, SNPs has been found to be related to susceptibility to gastric cancer 36, lung cancer 37, head and neck squamous cell carcinoma 38, breast cancer 39, colorectal cancer 40, and other malignant tumors. As illustrated in **Figure 1**, Linc00673, as a tumor suppressor, can increase the interaction between PTPN11 and PRPF19. However, PRPF19 belongs to the E3 ubiquitin ligase and is extremely important in promoting the degradation of PTPN11. Therefore, it inhibits the downstream carcinogenic SRC-EPK pathway and activates the STAT1-dependent tumor suppressor pathway 41.

Current studies on SNPs of Linc00673 mainly focus on rs11655237. Reportedly, rs11655237 is located on exon 4 of Linc00673 11, 41 and 6 SNPs are involved in high-linkage disequilibrium with rs11655237 41. This SNP increases the susceptibility of the population to pancreatic cancer 11, 41, gastric cancer 42, hepatoblastoma 43, neuroblastoma 44, 45, and cervical cancer 46, 47 in an allele-specific manner. rs11655237 regulates the expression level of Linc00673 by forming a region that miR-1231 recognizes and binds with 41, 42, 46, 47. Indeed, the formation of this miRNA-recognition region can be attributed to the conversion of the G allele to the A allele at rs11655237. Risk variant A blocks the normal function of Linc00673 by increasing the binding efficiency to miR-1231. As a result, Linc00673 is downregulated, PTPN11 is accumulated, and an oncogene IFNAR1 is upregulated. These changes promote the proliferation of pancreatic cancer cells and the growth of cervical tumors 41, 47. Zhu et al. found that, among patients with cervical cancer, those carrying the A allele of rs116552337 showed worse-rate overall survival than those carrying the G allele of rs11655237 47. In addition, according to Haplo Reg v2, rs11655237 detected significant DNase hypersensitivity in multiple cancer cell lines and binding to transcription factors, including P300, FOXA1, FOXA2, and DNA repair protein RAD21 11, 48. However, this observation has not been specifically reported in the literature, which suggests that SNPs may affect the regulation of disease development by interfering with the binding of Linc00673 to transcription factors.

### Interacts with RNA-binding proteins to regulate tumor development

**Figure 2:**
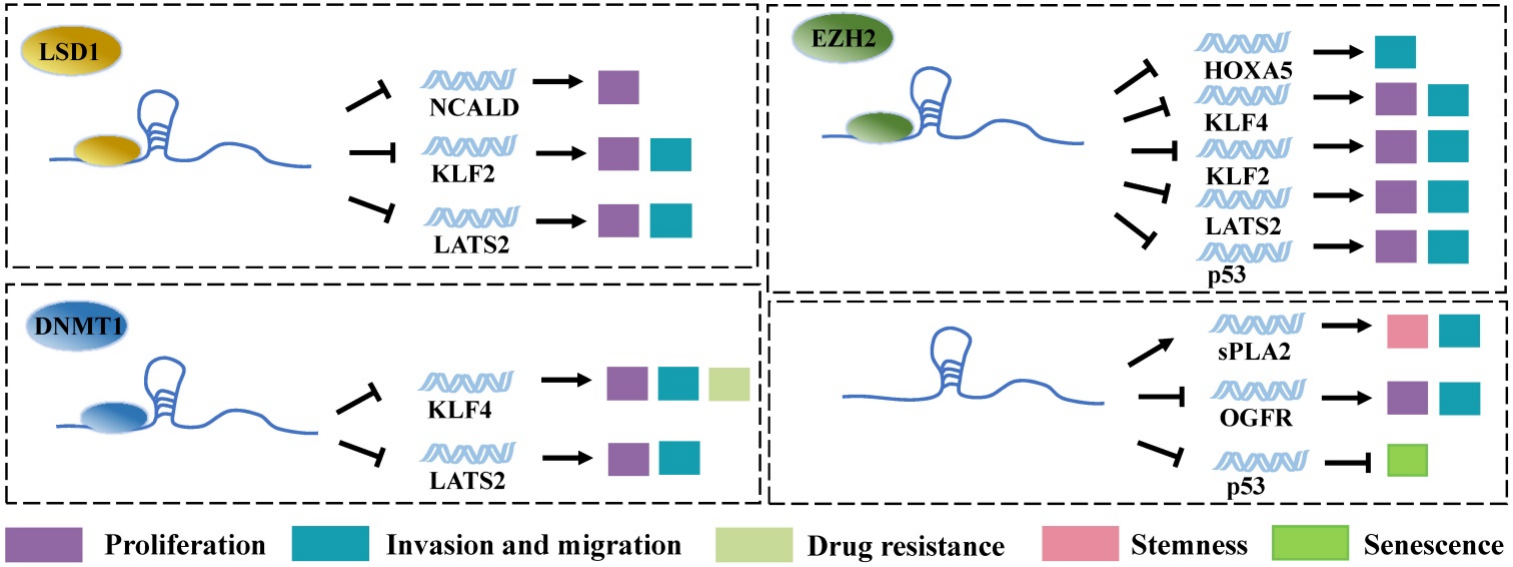
Linc00673 binds to RBPs. Linc00673 regulates the expression of downstream target gene and regulates the proliferation, migration, invasion, drug resistance, stemness, and senescence of tumor cells by binding to LSD1, E2H2, and DNMT1.

Among proteins that can be regulated by LncRNAs, transcription factors are the key ones in transcriptional regulation. In most of the typical models, gene expression control is believed to be mediated by DNA-binding proteins (DBPs). The activation of DBPs is usually regulated by signaling pathways, and their DNA-binding ability is related to sequence specificity. In contrast, RNA-binding proteins (RBPs) are often believed to be co-transcribed or post-transcribed 49. LncRNAs exhibit different functional domains on their long sequences, which can bind to corresponding RBPs and thereby affect the stability, intracellular localization, and functioning of the respective proteins. Notably, LncRNA plays important biological functions in transcriptional regulation, epigenetics, and selective splicing through these RBPs 50. Acting as transcription factors, SP1 19, E2F1 51, and YY1 22 can bind directly to the promoter region of Linc00673 mRNA, which significantly increases the expression level of Linc00673. Moreover, the transcribed Linc00673 can bind to RBPs such as histone demethylase lysine specific demethylase 1 (LSD1) 13, 19, enhancer of Zeste Homolog 2 (EZH2) 18, 19, 24, 52, and DNA methyl-transferase 1 (DNMT1) 18, 24. These RBPs are involved in epigenetic modifications, particularly in the methylation of genes. Thus, Linc00673 can regulate the expression of downstream genes through these 3 proteins and exert the functions of tumorigenic genes. For example, Huang et al. applied bioinformatics predictions and performed radioimmunoprecipitation assays and found that Linc00673 can bind to LSD1 and EZH2 19. Moreover, EZH2 and LSD1 were found to bind directly to the promoter regions of tumor suppressor genes LATS2 and KLF2, which induces the H3K27 trimethylation or H3K4 demethylation of these genes and downregulates their expression. This event ultimately promotes the proliferation, invasion, and migration of gastric cancer cells. EZH2 is a member of Polycomb Repressive Complex 2 (PRC2). This complex can inhibit the transcription of target genes by methylating the H3K27me of histone H3 53. DNMT1 is also a protein that is widely involved in gene methylation modification. Therefore, Linc00673 can downregulate the expression of tumor suppressor genes such as HOXA5 52, KLF4 18, 26, and p53 24 through EZH2 and DNMT1. A study on lung cancer revealed that, in addition to functioning through binding proteins, Linc00673 can directly bind to the 3' untranslated region of TP53, reduce the level of p53 transcription, and cancel p53-mediated cell cycle arrest, which altogether result in the generation of cellular senescence bypass 51. This points suggest that Linc00673 possesses certain special sequences that can directly bind to mRNA for the regulation of the expression of the target gene without needing RBPs. LSD1, another important enzyme involved in epigenetic modifications, can also assist Linc00673 in promoting tumor progression via downregulating the expression of the tumor suppressor gene NCALD 13. In addition, the high expression level of Linc00673 is also associated with the downregulation of the expression of opioid growth factor receptor (OGFR) 27 and the upregulation of the expression of secretory phospholipase A2 (spla2) 30, although the molecular mechanisms involved in these events remain unexplored (**Figure 2**).

### Formation of a competitive endogenous RNA (ceRNA) network with microRNAs to regulate tumor development

**Figure 3:**
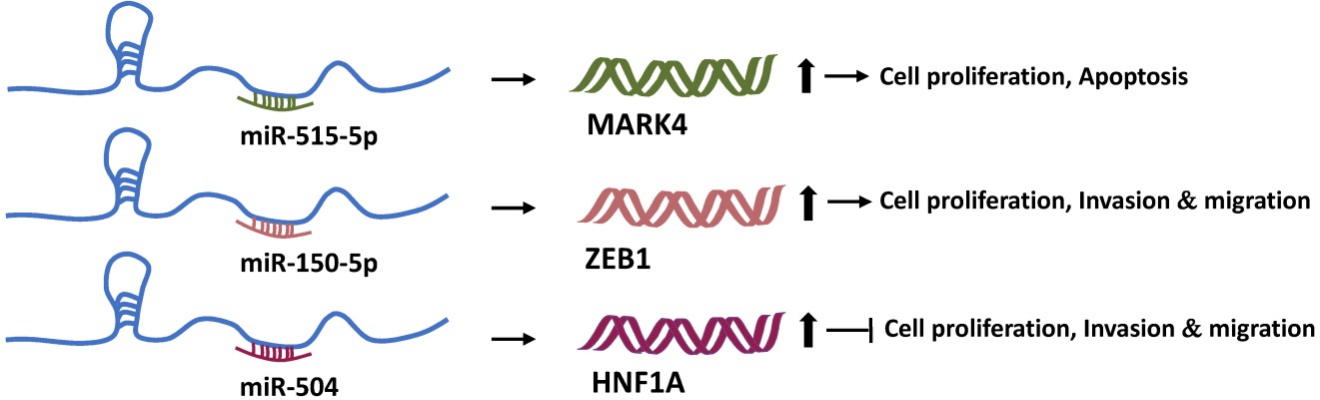
Linc00673 and mRNA competitively bind to miRNAs. Linc00673 can adsorb miR-515-5p, miR-150-5p, and miR-504, and upregulate the expression levels of MARK4, ZEB1, and HNF1A mRNA. Finally, it promotes tumor progression or inhibits tumor progression.

We discussed how Linc00673 regulates the transcription of downstream genes with the help of RBPs. Undoubtedly, LncRNAs can also bind to other RNA molecules based on the principle of complementary base pairing. In 2011, Pandolfi et al. of the Harvard Medical School proposed the hypothesis of ceRNA. According to this hypothesis, transcripts such as coding RNAs, pseudogenes, and non-coding RNAs can regulate each other's expression by competitively binding to “microRNA response elements” 54. This microRNA-mediated communication method constitutes a huge regulatory network among genes. MicroRNAs (miRNAs) are known to negatively regulate the target gene expression 55. Therefore, Linc00673 can regulate the expression of other genes through miRNAs. A past study found that Linc00673 can sponge-absorb miR-515-5p, relieve the inhibition of miR-515-5p on microtubule affinity regulating mitogen-activated protein kinase 4 (MARK4), and activate the hippo signal pathway participated by MARK4 in order to promote the proliferation of breast cancer cells 22. In addition, Linc00673 can also competitively bind to miR-205 23, miR-150-5p 14, and miR-504 16. Moreover, through miR-150-5p and miR-504, Linc00673 can regulate the expression of ZEB1 14 and HNF1A 16, respectively, and then exert tumor-promoting or anti-tumor activity (**Figure 3**).

### Binding to steroid receptors to regulate tumor progression

**Figure 4:**
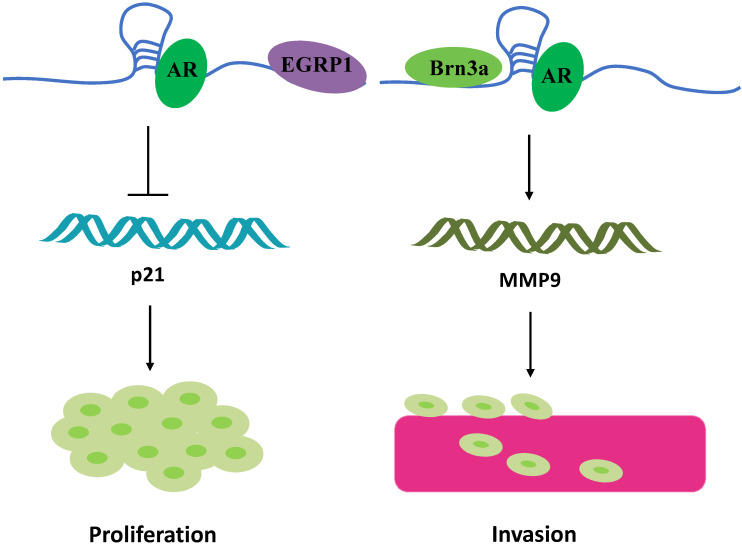
Linc00673 binding to steroid receptors. Linc00673 combines with AR to form trimers with EGRP1 and Brn3a, respectively, and ultimately promotes the proliferation and invasion of melanoma cells.

Another research direction that has attracted attention is the potential “gender preference” of Linc00673. For instance, Schmidt et al. found that Linc00673 is highly expressed in melanoma cells 10. Meanwhile, they found a highly conserved sequence region on Linc00673 that is similar to SRA1. Brain-specific homeobox protein 3a (Brn3a) and androgen receptor (AR) bind to this conserved sequences and the adjacent sequences of Linc00673, respectively. The resultant 3-polymer model eventually promotes melanoma invasion at the transcriptional level by regulating the expression of matrix metalloproteinases 9 (MMP9) (Figure 4) 10. Linc00673 can also recruit AR to the EGR1 genome-binding site, which is a zinc finger transcription factor, and then inhibit the expression level of p21 mRNA independent of p53, which in turn ultimately promotes the proliferation of melanoma cells (**Figure 4**) 56. Therefore, blocking the combination of AR and Linc00673 can limit the invasion of melanoma mediated by Linc00673 57. Although Linc00673 induces AR in a ligand-independent manner, the association of AR to melanoma invasion may partially explain why male melanoma patients experience more metastasis and lower survival than the corresponding female patients. SRA1 is a bifunctional gene that produces both functional RNA SRA and corresponding proteins 58. SRA is a non-coding RNA that specifically coactivates the transcriptional activity of steroid receptors 59, 60. In line with its function, the expression of SRA is upregulated in steroid hormone-responsive tissue (such as in the breast, uterus, and ovary) tumors as compared with that in the normal tissues 61, 62. Owing to the special presence of steroid hormone response tissues and the conserved sequence similar to SRA on Linc00673, Linc00673 possibly participates in the occurrence and development of cancers of the female reproductive system. Breast cancer has different subtypes, classifiable as ERα+, ERα-, and HRE2+. Abdul-rahman et al. performed a hierarchical analysis of the Affymetrix U133 Microarray data and the TCGA data set for breast cancer and found that, as compared with ER+ patients, the expression of Linc00673 in ER- patients was higher, while the high expression of LINC00673 in ER- patients was associated with adverse outcomes 21. In addition, Linc00673 plays the role of an oncogene in ovarian cancers 27. Based on the sequence specificity of Linc00673 and relevant past reports, Linc00673 is speculated to be involved in the regulation of tumor development through sex hormone receptors.

### Involvement in the regulation of the epithelial-mesenchymal transition (EMT) of tumors

**Figure 5:**
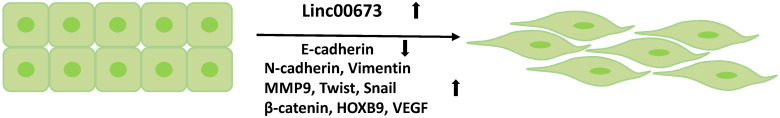
Linc00673 promotes the EMT process of tumor cells. The high expression of Linc00673 promotes the downregulation of E-cadherin and the upregulation of mesenchymal markers in tumor cells.

The EMT is associated with tumor cell invasion and metastasis. During the EMT, the expression of epithelial markers is downregulated, while that of the mesenchymal markers is upregulated. Linc00673 can promote the EMT process in tumor cells 20, 23, 25 (**Figure 5**) by downregulating the expression of E-cadherin and upregulating the expression of mesenchymal markers, such as N-cadherin, vimentin, snail 14, and MMP-9 63, 64. In addition, multiple signaling pathways are involved in the regulation of this process. Guan et al. found that Linc00673-v4-the most abundant transcription of Linc00673 in lung adenocarcinoma cells and a molecular scaffold-enhanced the interaction between DDX3 and CK1e proteins, activated the WNT/β-catenin signaling pathway, and increased the expressions of nuclear β-catenin, VEGF, Twist, HOXB9, and MMP9, which together increased the aggression of lung adenocarcinoma 64. In addition, Linc00673 is also involved in the activation of the PI3K/AKT signaling pathway to promote glioma cell migration and invasion 65. The PI3K/AKT signaling pathway has been implicated in the regulation of EMT in a variety of tissues, including gliomas 66-68. Although the research reports of Linc00673 in promoting tumor progression through EMT has been well discussed, it is undeniable that Linc00673 is tissue specific. For example, in pancreatic ductal carcinoma, Linc00673 acts as a tumor suppressor gene. Arnes et al. demonstrated that silencing of Linc00673 can significantly increase the expression of MET. The upregulated MET promotes the downregulation of the epithelial markers of pancreatic cancer cells (FOXA1 and CDH1) and the upregulation of vimentin expression, which eventually promotes the EMT process and increases tumor cell migration 9.

## Conclusion

Linc00673 is upregulated in several tumor tissue types; this process is accompanied with complex molecular regulatory mechanisms. Linc00673 interacts with RBPs, regulates the expression level of downstream target genes at the epigenetic level, and absorbs miRNA to participate in the construction of competitive network relationships among genes. In addition, it also participates in the regulation of SRC-EPK, WNT/β-catenin, PI3K/AKT, p53, and EMT-related signaling pathways. Finally, in such a situation, the tumor cell proliferation, migration, invasion, drug resistance, stemness maintenance, and other functions get modified. Because of its conserved sequence similar to SRA, the interaction between Linc00673 and the steroid receptor implies that an imbalanced expression of Linc00673 has a certain gender preference. This point raises a question of whether Linc00673 participates in the pathogenesis of lupus erythematosus, thyroiditis, and prostate diseases in addition to neoplastic diseases. This question deserves our consideration and further exploration. Research in prostate cancer indicates that Linc00673 is associated with resistance to paclitaxel chemotherapy drugs 26. Moreover, Linc00673 was found to be involved in mediating the permeability of the blood-tumor barrier in the glioma as well as in promoting the killing effect of the drugs on the tumor cells 69. Accordingly, Linc00673 may be used as a molecular target to prevent drug resistance and to provide treatment inspiration for patients resistant to chemotherapy drugs. Indeed, SNPs can also affect patients' responsiveness to chemotherapy drugs 70, 71. Thus, whether SNPs of Linc00673 affects patients' resistance to drug treatment also needs to be considered in the future research.

Although Linc00673 is involved in the regulation of tumor progression, the biological functions of linc00673 remain to be explored. The diversification of the structure allows an RNA to perform multiple functions. For example, the *maternally expressed gene 3* (MEG3) inhibits tumor growth, and all MEG3 RNA subtypes contain 3 different secondary folding motifs M1, M2, and M3 72. Han et al. evaluated the minimum free energy of the LncRNA AFAP1-AS1 structure and found that a stem-loop structure formed at the 91-1190 nt locus can interact with the RBP 73. However, no researcher has focused on the structure of linc00673 until date. Therefore, it is extremely important to explore the biological functions of linc00673 in diseases by studying the structure of linc00673.

In addition to the verification of the expression level of Linc00673 in tissues, its presence in patients' blood offers a great potential to consider it as a biomarker in the real-time and dynamic monitoring of the tumor development. The proposed approach shall provide a new insight to improved clinical auxiliary diagnosis, prognosis prediction, efficacy evaluation, and individualized diagnosis and treatment. However, presently, the studies on Linc00673 are limited. The question remains whether it is present in a patient's urine, saliva, and other body fluids in addition to the peripheral blood. Moreover, whether Linc00673 participates in tumor regulation through the molecular signaling pathways related to exosomes is also worth investigating. In fact, the content of circulating tumor nucleic acid in the blood is extremely low, making it a technical challenge toward separating the free-circulating nucleic acid from the peripheral blood for enrichment proposes 74. In addition, the level of circulating nucleic acid secreted by early tumors is low and hence cannot be monitored accurately 75. Therefore, a convenient, economical, and sensitive detection method is necessary to promote the application of linc00673 as a “liquid biopsy” in clinical practice.

In general, as a newly discovered non-coding RNA, the precise mechanism of upstream regulation and downstream signal transduction of Linc00673 remains to be studied systematically and confirmed through in-depth research for the purpose of promoting its clinical application.

## Figures and Tables

**Table 1:** Clinical significance of Linc00673 in various cancer types

Cancer type	Expression	Clinicopathological features	References
Lung cancer	Upregulated	Tumor size, lymphatic metastasis, TMN stage, diagnostic biomarker, ADC/SCC, poor prognosis	13-15, 30, 52, 64
Pancreatic cancer	Upregulated	Clinical stages, pathological metastasis, lower survival percent	17
Gastric cancer	Upregulated	Tumor size, advanced pathological stage, lymph node metastasis, poor prognosis, distant metastasis, TNM stage	18, 19
Mammary cancer	Upregulated	Lymph node metastasis, clinical stage, tumor size ki67 status, shorter OS	20, 22
Liver cancer	Upregulated	Advanced clinical stage, lower OS	23
Thyroid carcinoma	Upregulated	Tumor size, lymph node metastasis, worse prognosis	24, 25
Prostatic cancer	Upregulated	Tumor size, TNM stage, lymph node metastasis	26
Ovarian cancer	Upregulated	Histological subtype, FIGO stage, lymph node metastasis, shorter PFS, shorter OS, independent prognostic factors	27
Tongue cancer	Upregulated	Tumor size, invasion muscles of tongue, higher TNM stage, relapse, poor prognosis	28
Colorectal cancer	Upregulated	TNM stage, tumor size, regional lymph node metastasis, distant metastasis, lower OS	29

Abbreviations: ADC/SCC, adenocarcinoma/squamous cell carcinoma; OS, overall survival; PFS, progression-free survival; FIGO, International Federation of Gynecology and Obstetrics.

## Abbreviations

LncRNAs: Long non-coding RNAs
SRA1: steroid receptor RNA activator 1
SLNCR: SRA-like non-coding RNA
SNPs: single nucleotide polymorphisms
TCGA: The Cancer Genome Atlas
GWAS: Genome-wide association study
DBPs: DNA-binding proteins
RBPs: RNA-binding proteins
LSD1: lysine specific demethylase 1
EZH2: Enhancer of Zeste Homolog 2
DNMT1: DNA methyl-transferase 1
PRC2: Polycomb Repressive Complex 2
OGFR: opioid growth factor receptor
spla2: secretory phospholipase A2
MicroRNAs: miRNAs
MARK4: mitogen-activated protein kinase 4
Brn3a: Brain-specific homeobox protein 3a
AR: androgen receptor
MMP9: matrix metalloproteinases 9
EMT: Epithelial-Mesenchymal transition
MEG3: maternally expressed gene 3
